# Chronic Obstructive Pulmonary Disease and Dysphagia: A Synergistic Review

**DOI:** 10.3390/geriatrics5030045

**Published:** 2020-08-24

**Authors:** Ting-fen Lin, Samantha Shune

**Affiliations:** Communication Disorders and Sciences Program, University of Oregon, Eugene, OR 97403, USA; sshune@uoregon.edu

**Keywords:** COPD, deglutition, dysphagia, mind body breath, person-centered care, quality of life, respiration

## Abstract

Chronic obstructive pulmonary disease (COPD) is a leading global cause of death and disability. The literature has previously established clear physiological characteristics of COPD-related dysphagia (swallowing difficulties). However, COPD and dysphagia are both also intimately tied to breathing and contribute to a cascade of secondary physio-psycho-emotional sequalae, such as COPD exacerbation, anxiety, depression, increased economic burden, social isolation, and decreased quality of life. Further, the collective impact of these comorbidities may magnify disease impact, resulting in a downward spiral of well-being. Thus, the clinical relevance of COPD’s and dysphagia’s frequently occurring and overlapping sequelae cannot be overlooked, as the disease-related burden of both disorders is deeply rooted in the presence of concomitant physiological and psycho-emotional consequences. The current review explores the complex network of interactions between COPD, dysphagia, and their outcomes, framing this relationship within a mind-body-breath framework. Ultimately, we propose a model that more comprehensively captures the constellation of interrelated disease characteristics and consequences, highlighting a need for researchers and healthcare providers to consider disease impact more broadly in order to maximize treatment outcomes.

## 1. Introduction

Chronic obstructive pulmonary disease (COPD) is a life-threatening lung disease that is characterized by chronically obstructed airflow in the lungs, with a prevalence that increases dramatically with increasing age [1]. The mortality associated with this “common, preventable and treatable disease” [2] nearly doubled from 1970 to 2000 [3]. This highly prevalent disease is expected to be the third-leading cause of death [4] and a top ten cause of disease burden by 2030 [5]. 

COPD’s extensive disease-related burden across the physical, psycho-emotional, and economic domains has been readily profiled. COPD is linked to a number of health consequences including increased risk of mortality, COPD exacerbations, anxiety/depression, economic burden, and aspiration pneumonia, more frequent hospital (re)admissions, increased length of hospital stays, and decreased quality of life [6,7,8,9,10,11]. The relationships between the disease itself and its related sequalae are complex, supporting the need to more broadly understand both the factors influencing COPD and the disease’s consequences. For example, a recent systematic review demonstrated the various interactions between anxiety, depression, and acute COPD exacerbations and resultant hospital admissions or re-admissions [12]. At the most linear level, it would appear that increased anxiety and depression can lead to increased exacerbations and subsequent hospitalizations, which in turn contribute to a decreased ability to cope, further increasing anxiety and depression. However, the relationship between these factors was found to be further multifaceted. Increased dyspnea, length of hospital stay, risk of mortality, and loss of autonomy as well as decreased quality of life and self-esteem all mediated the relationship between COPD and its psycho-emotional comorbidities and interacted with each other. Further, individuals with COPD and concomitant anxiety/depression are at greater risk as compared to those without concomitant anxiety/depression not only of developing COPD exacerbations, but also of needing to utilize medical expenses [9]. The influence of such economic burden is likely to reverberate across an individual’s daily life, negatively impacting stress, anxiety, depression, and quality of life. The consequences of long-term anxiety and depression are substantial, further decreasing quality of life [13,14]. Ultimately, the physiological impairments associated with COPD (e.g., dyspnea, exacerbations) have a cascading effect on the psycho-emotional aspects of life (e.g., anxiety, depression) and vice versa, dramatically increasing healthcare expenditures and reducing quality of life. Further, the prevalence of comorbidities (e.g., arterial hypertension, diabetes, anxiety and depression) in individuals with COPD is high (up to 80%) [15] suggesting an additional layer of physio-psycho-emotional variables that may interact with and contribute to COPD’s influence on an individual, particularly in the context where physical activities and social relationships are limited by their lung dysfunction. 

One contributor to COPD disease-related burden is dysphagia. While the exact prevalence of dysphagia among individuals with COPD is unknown [16], COPD disrupts the typical coordination between the swallowing and respiratory systems, leading to impairments and inefficiencies in the swallowing process [17,18,19]. Dysphagia symptomatology is present even in individuals with mild COPD [20], although swallowing difficulties are not always recognized or self-reported as an issue [21]. Among nursing home residents, COPD has been found to be the second strongest predictor for aspiration pneumonia, which is also linked to dysphagia [22]. Unfortunately, the compromised breathing-swallowing pattern observed in individuals with COPD jeopardizes airway protection, leading to increased risk of aspiration and resultant pulmonary consequences [19]. Individuals with dysphagia experience increased risk of mortality as compared to individuals without dysphagia [23,24]. Thus, the interplay between COPD and dysphagia is one potential mechanism contributing to COPD’s high rates of mortality.

Dysphagia is also associated with increased morbidity, including dehydration, malnutrition, increased psycho-emotional burden, and decreased quality of life [25,26,27,28,29,30]. Individuals with dysphagia are more likely to experience a longer hospital stay, higher hospital bills, and a greater likelihood of needing a post-discharge medical placement [23]. Inpatient hospital costs for individuals with dysphagia have been found to be 40–60% higher than costs for individuals without dysphagia, a significant economic burden [31]. Dysphagia can also lead to social isolation and decreased patient and caregiver quality of life, negatively impacting psychological well-being, particularly as related to food experience and social life [32,33,34]. Thus, similar to COPD and other chronic illnesses, dysphagia influences a person’s overall physiological and psychological health. Therefore, it is probable that dysphagia is also a strong contributor to the increased morbidity in individuals with COPD. 

It is clear that COPD and dysphagia, likely both individually and jointly, impact an individual beyond the physiological level alone, negatively influencing overall psycho-emotional well-being. Thus, to maximize treatment outcomes and improve quality of life, COPD and COPD-related dysphagia may be better understood through more explicit characterization of the interactions between the physiological (breath and body) and the psycho-emotional (mind). However, previous research has generally focused on either physiology or psycho-emotional consequences. This paper first introduces the mind-body-breath connection as a guiding framework and then integrates the literature pertinent to the physiological and psycho-emotional characteristics and consequences of COPD and dysphagia, highlighting their key areas of overlap. Throughout, the mind-body-breath connection will be illustrated as a model to draw on for future management and research directions. 

## 2. Mind-Body-Breath Feedback Loop

**Figure 1:**
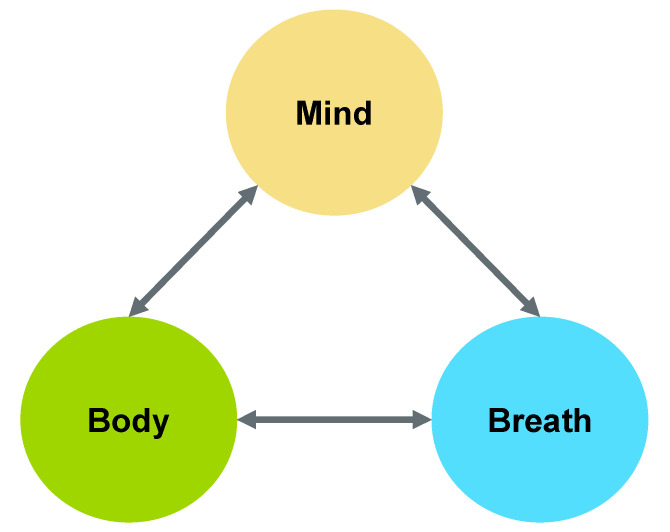
Mind-body-breath feedback loop.

While both COPD and dysphagia are physiologically-based diseases, their consequences extend far beyond physiology. What is less clear, however, is whether the relationship between the physiology of the disease and the non-physiological consequences is multidirectional. Psycho-emotional consequences may actually lead to a downward spiral of the disease, negatively impacting the impaired physiology, which may further magnify the psycho-emotional consequences of the disease in a more cyclical manner. Support for such a relationship is observed in studies of the mind-body-breath connection, which suggests that the actions of the mind, body, and breath are all interrelated and interdependent (see Figure 1) [35,36,37,38]. For example, this multidirectional relationship between physiology (body/breath) and psychology (mind/breath) is apparent under stressful conditions. A stressful state of mind can lead our nervous system into fight-or-flight mode. This activation of the parasympathetic nervous system is characterized by a faster respiratory rate, an alteration of breathing patterns, and a heightened alert state. 

The mind-body-breath connection can be similarly applied to dysphagia, particularly in the case of COPD. For example, dysphagia has a strong physiological base, including deficits in the oral, pharyngeal, and/or esophageal stages of swallowing. Optimizing or compensating for these physiological impairments are the common foci of treatment [39,40]. Yet, dysphagia can also be accompanied by anxiety and depression resulting from the swallowing impairments [27,28,41,42]. When individuals are experiencing negative emotions, such as depression, they often do not have the same interest in food intake [43], showcasing a bidirectional influence between mind and body. Further, breath is a vital source of energy in keeping the body alive, but requires alteration during eating to prevent food/liquids from entering the lower airway as evidenced by a short period of protective cessation of respiration. For a patient who is air hungry—the sensation of not having enough oxygen—particularly when given a compromised pulmonary status (e.g., a patient with COPD), the body is forced to open the airway to breathe for survival despite the need to close the airway during swallowing. In this situation, two opposing systems (i.e., open vs. closed airway) often lead to airway protection issues and consequent aspiration. As a result, aspiration pneumonia may develop and the patient’s respiratory load becomes even heavier. Clearly, an intimate relationship exists between body and breath. More subtly at the mind-breath level, there exists a bidirectional relationship between respiration and emotion. Previous research has suggested that respiration can influence emotion, and vice versa [36,37,38]. For example, trained breathwork can improve anxiety, stress, depression, positive affect, mental health, life satisfaction, and social connectedness [44,45]. In the case of COPD, compromised pulmonary status (breath) is often associated with anxiety (mind), which exacerbates the respiratory system and results in further increased anxiety [46]. Thus, better understanding the physiological impairments, psycho-emotional sequelae, and interactions between the physiology and psycho-emotional aspects of COPD-related dysphagia appears crucial for optimizing clinical outcomes.

## 3. Dysphagia in COPD—Primary Physiological Impairment

The swallowing system provides an avenue for meeting nutritional, hydrational, and, at times, medical needs. The musculature and associated physiology also act as a gateway in protecting the lower airway from aspiration. One mechanism of swallowing-related airway protection is laryngeal adduction, or a short cessation of respiration during swallowing. Intact laryngopharyngeal sensitivity and triggering a timely laryngeal adductor reflex is also important for airway protection [47]. When the swallow-respiratory coupling or sensitivity threshold is compromised, individuals are at high risk of pulmonary consequences (e.g., aspiration pneumonia, silent aspiration). Other dysphagia characteristics across the oral and pharyngeal systems can also impede safe and healthy swallowing function in COPD.

### 3.1. Respiratory-Swallowing Discoordination

Respiration patterns play a key protective role in swallowing in order to prevent food and liquids from entering the lower airway. There are four potential respiratory-swallow patterns, depending on whether the action of swallowing is immediately preceded and followed by inhalation or exhalation: (1) expiration-swallow-expiration, (2) expiration-swallow-inspiration, (3) inspiration-swallow-expiration, and (4) inspiration-swallow-inspiration. The first pattern, where the swallow is sandwiched in between expiratory flows, is the most predominant phase coupling pattern observed in normative data from healthy individuals [48,49]. During the expiration phase of respiration, the larynx is positioned in a paramedian position. This more adducted positioning of the arytenoids and true vocal folds before and after the swallow is thought to better protect the airway as compared to the abducted position observed during inhalation. Additional airway protection advantages of having an exhalation before and after the swallow include: (1) during exhalation, the diaphragm is relaxed with the larynx elevated (vs. during inspiration where the diaphragm contracts with the larynx lowered) [50]; and (2) swallowing followed by exhalation can help expel any penetrated material out of the airway [51].

However, this optimal coupling of the breathing and swallowing cycles is disrupted in the presence of COPD, even when the disease is stable [19]. The inspiration-swallow-expiration pattern is more commonly seen in individuals with COPD [52]. Both stable and exacerbated states of COPD are associated with increased respiratory rate in individuals with COPD as compared to healthy younger and older adults [18]. As their respiratory rate increases, these individuals also present with more inspirations before the swallow [52]. Individuals with COPD also present with an inspiration-swallow-inspiration pattern more often during exacerbations [18]. Further, increased respiratory rates and lower baseline oxygen saturations are associated with compromised swallowing-related airway protection (i.e., penetration or aspiration); increased respiratory rates at baseline can be predictive of airway invasion [52]. Tachypnea and/or dyspnea clearly modify the intimate coupling between respiration and swallowing. Disruption in coordination, particularly inhaling before and/or after swallows, ultimately decreases airway protection during swallowing, increasing the risk of aspiration and subsequent COPD exacerbations. Unfortunately, the resultant exacerbations in turn further disrupt swallowing coordination. This exemplifies the dynamic interplay between the breath and body in COPD-related dysphagia.

Another factor influencing respiratory-swallowing coordination and resulting swallow safety/efficiency is lung volume. Swallow initiation generally occurs within a narrow range of lung volumes (~44% vital capacity) [53]. A systematic review and meta-analysis concluded that, depending on the type, volume, and method of bolus consumption, healthy individuals swallow with lung volumes varying within the tidal volume range [54]. Individuals with COPD have been found to swallow at significantly lower lung volumes and at a lower percentage of vital capacity than healthy older adults [55]. The lower lung volumes during swallowing support the hypothesis that the respiratory-swallowing discoordination observed in individuals with COPD may result, at least in part, from decreased feedback from the pulmonary stretch or subglottal pressure receptors to the respiratory central pattern generator. These lower lung volumes were also found to be associated with longer pharyngeal durations, which may have been related to air hunger. Further, while individuals with COPD were more likely than healthy older adults to exhibit a post-swallow inhalation, they presented with this less often when swallowing at higher lung volumes than when swallowing at lower volumes.

### 3.2. Oropharyngeal Swallowing Impairments

Oropharyngeal swallowing physiology among individuals with COPD has been characterized across multiple studies [47,56,57,58,59,60,61]. Physiological impairments in this population include: reduced tongue control, reduced anterior-posterior tongue movement, reduced lingual stabilization, reduced tongue strength, delayed pharyngeal swallow, reduced tongue base retraction, slowed/delayed vestibule closure, reduced laryngeal elevation, abnormal swallow reflex, laryngeal penetration, (silent) aspiration, and cricopharyngeal dysfunction. More general dysphagia-related complaints and gastroesophageal reflux symptoms are more prevalent among the COPD population as compared to controls without pulmonary complaints [57]. Of concern, individuals experiencing acute exacerbation are more likely to demonstrate an impaired swallow reflex as compared to those with stable COPD [60]. Further, individuals with COPD who demonstrate an abnormal swallow reflex also present with a significantly higher occurrence of exacerbations, highlighting the bidirectional body-breath connection. In other words, when pulmonary status is compromised, swallow physiology (e.g., swallow response, respiratory-swallowing coordination) is affected, and vice versa.

Relatedly, a review of the literature revealed a relationship between dysphagia and COPD exacerbations in patients with impaired respiratory-swallow patterns [62]. Further, individuals with COPD who demonstrate an abnormal swallow reflex present with a significantly higher occurrence of exacerbations [61]. Thus, the presence of dysphagia can serve as a risk factor for COPD exacerbations, which can contribute to dysphagia symptomatology—further support for the bidirectional influence of body and breath.

## 4. Dysphagia in COPD—Primary Psycho-Emotional Sequalae

The psycho-emotional effects of both COPD and dysphagia are widespread. These effects both stem from and contribute to physiological impairments. Unfortunately, the non-physiological comorbidities likely impact each other as well as further influence the disease-related physiology. 

### Shared Psychoemotional Impact

COPD affects not only physiological, but also psycho-emotional well-being. Apart from the well-documented social isolation experienced by individuals with COPD [63,64], there is a high prevalence of anxiety (10% to 100%) and depression (7% to 79.1%) among these individuals [6,13]. Based on data from the 2004 Health and Retirement Survey, respiratory symptoms and difficulty walking several blocks are both major risk factors for depressive symptoms in individuals with COPD [65]. Relatedly, those with COPD are reportedly more likely to exhibit depressive symptoms as compared to those with other common chronic illnesses, like coronary heart disease, stroke, hypertension, diabetes, arthritis, or cancer. Additional studies using standardized clinical interviews and questionnaires also support the relationship between COPD and emotional disturbances like anxiety and depression, or a linkage between the body-breath and the mind [66]. 

Interestingly, while COPD increases the risk of long-term anxiety and depression, the resulting psycho-emotional comorbidities, in turn, also increase the risk of disease exacerbations [14,67,68]. Negative affect is noted to amplify the perception of dyspnea in individuals with COPD [69]. Individuals with comorbid anxiety and depression experience a higher incidence of frequent exacerbations (≥2/year; 73.8%) than those without (50.5%) [10]. A three-year longitudinal study suggested that one in four patients demonstrate persistent depressive symptoms and that those with depression undergo more COPD exacerbations [67]. Additionally, depression serves as a risk factor for acute exacerbation of COPD, in the short-term (30-day and 90-day) as well as for up to one-year readmission [70]. Together, these findings highlight the more nuanced relationship between negative emotions (the mind) and COPD disease status (the body and breath). Although the exact mechanisms underlying this relationship between COPD, exacerbation, and anxiety/depression are not clear, a growing body of research demonstrates that the relationship between the body/breath and mind is multidirectional, supporting the need for a more comprehensive look at the disease [71].

Similarly, dysphagia in general is a known risk factor for social isolation, anxiety and depression across a variety of populations [27,28,32,41,42,72]. Among nursing home residents, swallowing difficulties have been found to be a significant risk factor for depressive symptoms [42]. Similar results have been observed among community-dwelling individuals; of those who reported the presence of dysphagia (vs. absence), the prevalence of clinical depression was 7% vs. 5%; anxiety 20% vs. 14%; and neuroticism 18% vs. 13% [27]. In fact, in the development of the swallowing quality of life questionnaire (SWAL-QOL), data indicated that “33% of these dysphagic patients would screen positive for major depression” [73]. Further, dysphagia has been found to be related to social isolation, such as in the setting of involvement in work-related activities [32]. More broadly, eating-related activities are linked to quality of social relationships and individual identify; eating-related difficulties can actually increase the impact of a chronic disease by further isolating an individual from their social networks [74,75,76,77]. Clearly a variety of psycho-emotional and social consequences of dysphagia coexist with the physiological burden of the dysphagia itself. Given the frequent presence of dysphagia among individuals with COPD [16], it is likely that dysphagia then contributes to the psycho-emotional impacts of COPD on these individuals. This interaction between body and mind, particularly as revealed through the increased risk of anxiety, depression, and social isolation, further supports the need to characterize and treat disease beyond physiology alone.

## 5. Interplay between the Secondary Sequalae of COPD and Dysphagia 

In light of the mind-body-breath connection, the combined consequences of these diseases are substantial; in 2010, an estimated $32.1 billion in national medical costs were attributed to COPD and its sequelae—a number that is expected to rise [78]. Significantly, the secondary sequalae, such as increased economic burden and decreased quality of life, are likely to result from the combination of the primary physiological and psycho-emotional impacts of both COPD and dysphagia [7,8,13,14]. For example, the consequences of long-term anxiety and depression in individuals with COPD are associated with increased functional disability and healthcare utilization, and decreased quality of life and survival. Health-related quality of life in individuals with COPD is further complicated and adversely affected by frequent exacerbations. Like any chronic disease, dysphagia has also been reported to be related to reduced quality of life. For example, quality of life indices are lower for individuals with head and neck cancer experiencing dysphagia as compared to general population norms [25]. Across a systematic review of 35 studies, oropharyngeal dysphagia was found to decrease health-related quality of life while oropharyngeal dysphagia interventions that resulted in decreased symptom severity led to improved health-related quality of life [26]. For example, individuals with dysphagia of varying etiologies showed improved health-related quality of life after undergoing device-facilitated isometric progressive resistance oropharyngeal therapy for eight weeks [79]. Conversely, other research has demonstrated that affective burden increases somatic complaints [80,81] and that despite improved swallowing function, the perception of improved quality of life may be more limited [82]. While these studies do not specifically target individuals with COPD, the findings support further exploration of symptom severity, intervention outcomes, and quality of life among individuals with COPD. The disease-specific comorbidities of COPD and dysphagia are likely to be intertwined, much like the physiological and psycho-emotional impairments and consequences. That is, the comorbidities of COPD and dysphagia contribute to one another, adding layers of disease burden that may ultimately feed back into the primary diseases themselves, worsening clinical outcomes. As the population of individuals with COPD and dysphagia continues to rise [4,83], a more comprehensive view of the two conditions and their subsequent outcomes is essential in order to better target management.

## 6. Discussion and Clinical Implications

**Figure 2:**
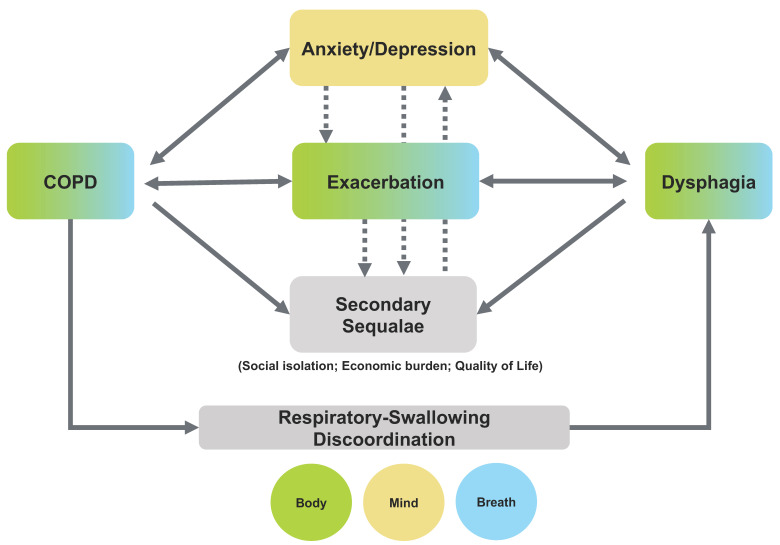
A model of the interplays between and across chronic obstructive pulmonary disease (COPD), dysphagia, and their related physio-psycho-emotional consequences within a mind-body-breath framework. The green boxes represent the body level, the tan boxes represent the mind level, and the blue boxes represent the breath level.

This review supports the existence of an intricate relationship between the physiological impairments and primary psycho-emotional consequences of COPD and dysphagia, as well as their secondary sequalae, that maps onto the mind-body-breath framework, as proposed in Figure 2. One entry point into the framework is COPD’s contribution to dysphagia via its influence on respiratory-swallowing coordination (bottom arrow in Figure 2). Both COPD and dysphagia are physiologically-based conditions, intertwining the body (green boxes) with the breath (blue boxes), either as a symptom (i.e., shortness of breath) or protection (i.e., swallowing respiratory cessation). Yet the comorbidities of COPD and dysphagia are widespread, influencing both the mind (tan boxes) as in anxiety/depression and the body/breath as in exacerbations. These comorbidities, in turn, loop back to influence the physiology of the diseases, particularly as the diseases progress. Both chronic conditions also share similar secondary sequalae related to more global effects on life. In addition to the direct impact of the diseases’ physiology on these secondary sequalae, the psycho-emotional consequences of anxiety/depression, and even the consequences of multiple exacerbations, subsequently affect the secondary sequalae also. These secondary sequalae may then magnify the psycho-emotional impacts of the disease, further contributing to the negative influence of COPD and dysphagia on individual well-being. This complex network of the relationships between disease and primary physio-psycho-emotional sequalae, as well as the intricate feedback loop of their secondary sequalae, requires greater research attention. As none of the boxes represented in Figure 2 occur in isolation, it also calls for a more comprehensive and systematic clinical lens to be used when working with individuals with COPD and/or dysphagia, particularly as the prevalence of these diseases is predicted to rise [4,83].

The well-documented presence of overlapping and interrelated COPD and dysphagia disease characteristics and consequences calls for a synergistic action plan. There is a need for increased attention from researchers and healthcare provides alike to identify and manage the comorbid psycho-emotional impacts of COPD and dysphagia, such as anxiety and depression, in order to optimize patient care. The cascading domino effect suggested in Figure 2 highlights the potential physiological, psycho-emotional, and economic risks of not doing so. While more research and resources in the co-management of COPD, dysphagia, and their comorbidities are needed, there are a number of items clinicians can integrate into their practices currently in order to better maximize patient well-being. During patient encounters, clinicians can explicitly ask about the broader experience of eating, focusing not only on swallowing difficulties, but also breathing-related concerns and resulting emotions. For example, how does the patient feel about the eating experience (e.g., stress, fear) and have these emotions changed over time? It may be beneficial to have a patient document their daily experience. The clinician and patient could then jointly explore the data to discover patterns related to the eating conditions in which shortness of breath is more noticeable or negative emotions are most magnified and potential strategies a patient is using that make swallowing and breathing easier. Engaging in open conversation can also allow the clinician to identify the need for referrals to additional healthcare providers, such as mental health professionals, strengthening the multidisciplinary team approach to COPD management. Importantly, these actions can better inform more individually tailored and person-centered management, expanding on the current conventional approaches such as diet modifications and rehabilitative or maneuver prescriptions. Targeted multi-system interventions, such as those that incorporate the mind-body-breath feedback loop (e.g., breath-based yoga/meditation), may also provide a novel direction for COPD-related dysphagia management and should be a target of future research work. 

## 7. Conclusions

The clinical relevance of COPD’s and dysphagia’s frequently occurring and overlapping sequelae cannot be overlooked, as the disease-related burden of both disorders is deeply rooted in the presence of concomitant physiological and psycho-emotional consequences. In integrating the literature, we proposed here a network of multidirectional relationships between and across the two diseases as well as their comorbidities within a mind-body-breath framework that may be useful in guiding future research and clinical practices (Figure 2). Given the multifaceted nature of both COPD-related and dysphagia-related influencers and sequelae, there clearly is an important need for a paradigm shift. We must move toward a more comprehensive management approach rather than our current standard-of-care approaches that treat the physiology of these conditions in isolation. Understanding the interplay between the physiology and psycho-emotional aspects of these diseases and their comorbidities, or the mind-body-breath connection, will ultimately maximize and benefit person-centered care.

## References

[1] Varmaghani M., Dehghani M., Heidari E., Sharifi F., Moghaddam S., Farzadfar F. (2019). Global prevalence of chronic obstructive pulmonary disease: Systematic review and meta-analysis. East. Mediterr. Health J..

[2] (2019). Global Initiative for Chronic Obstructive Lung Disease: Pocket Guide to COPD Diagnosis, Management, and Prevention. https://goldcopd.org/wp-content/uploads/2018/11/GOLD-2019-POCKET-GUIDE-FINAL_WMS.pdf.

[3] Diaz-Guzman E., Mannino D.M. (2014). Epidemiology and Prevalence of Chronic Obstructive Pulmonary Disease. Clin. Chest Med..

[4] (2019). World Health Organization (WHO). https://www.who.int/respiratory/copd/en.

[5] Mathers C.D., Loncar D. (2006). Projections of Global Mortality and Burden of Disease from 2002 to 2030. PLoS Med..

[6] Schneider C., Jick S.S., Bothner U., Meier C.R. (2010). COPD and the Risk of Depression. CHEST.

[7] Miravitlles M. (2004). Effect of exacerbations on quality of life in patients with chronic obstructive pulmonary disease: A 2 year follow up study. Thorax.

[8] Bentsen S.B., Miaskowski C., Rustøen T. (2013). Demographic and clinical characteristics associated with quality of life in patients with chronic obstructive pulmonary disease. Qual. Life Res..

[9] Dalal A.A., Shah M., Lunacsek O., Hanania N.A. (2011). Clinical and Economic Burden of Depression/Anxiety in Chronic Obstructive Pulmonary Disease Patients within a Managed Care Population. COPD J. Chronic Obstr. Pulm. Dis..

[10] Montserrat J.M., Godoy P., Marsal J.R., Barbe F., Pifarré J., Alsedà M., Ortega M. (2017). Overview of the Impact of Depression and Anxiety in Chronic Obstructive Pulmonary Disease. Lung.

[11] Zareifopoulos N., Bellou A., Spiropoulou A., Spiropoulos K. (2019). Prevalence, Contribution to Disease Burden and Management of Comorbid Depression and Anxiety in Chronic Obstructive Pulmonary Disease: A Narrative Review. COPD J. Chronic Obstr. Pulm. Dis..

[12] Pooler A., Beech R. (2014). Examining the relationship between anxiety and depression and exacerbations of COPD which result in hospital admission: A systematic review. Int. J. Chron. Obstruct. Pulmon. Dis..

[13] Hynninen K.M.J., Breitve M.H., Wiborg A.B., Pallesen S., Nordhus I.H. (2005). Psychological characteristics of patients with chronic obstructive pulmonary disease: A review. J. Psychosom. Res..

[14] Yohannes A.M., Willgoss T.G., Baldwin R.C., Connolly M.J. (2010). Depression and anxiety in chronic heart failure and chronic obstructive pulmonary disease: Prevalence, relevance, clinical implications and management principles. Int. J. Geriatr. Psychiatry.

[15] Fumagalli G., Fabiani F., Forte S., Napolitano M., Balzano G., Bonini M., De Simone G., Fuschillo S., Pentassuglia A., Pasqua F. (2015). INDACO project: COPD and link between comorbidities, lung function and inhalation therapy. Multidiscip. Respir. Med..

[16] O’Kane L., Groher M. (2009). Oropharyngeal dysphagia in patients with chronic obstructive pulmonary disease: A systematic review. Rev. CEFAC.

[17] Cvejic L., Bardin P.G. (2018). Swallow and Aspiration in Chronic Obstructive Pulmonary Disease. Am. J. Respir. Crit. Care Med..

[18] Shaker R., Li Q., Ren J., Townsend W.F., Dodds W.J., Martin B.J., Kern M.K., Rynders A. (1992). Coordination of deglutition and phases of respiration: Effect of aging, tachypnea, bolus volume, and chronic obstructive pulmonary disease. Am. J. Physiol. Gastrointest. Liver Physiol..

[19] Gross R.D., Atwood C.W., Ross S.B., Olszewski J.W., Eichhorn K.A. (2009). The Coordination of Breathing and Swallowing in Chronic Obstructive Pulmonary Disease. Am. J. Respir. Crit. Care Med..

[20] Ohta K., Murata K., Takahashi T., Minatani S., Sako S., Kanada Y. (2009). Evaluation of swallowing function by two screening tests in primary COPD. Eur. Respir. J..

[21] Garand K.L., Strange C., Paoletti L., Hopkins-Rossabi T., Martin-Harris B. (2018). Oropharyngeal swallow physiology and swallowing-related quality of life in underweight patients with concomitant advanced chronic obstructive pulmonary disease. Int. J. Chron. Obstruct. Pulmon. Dis..

[22] Langmore S.E., Skarupski K.A., Park P.S., Fries B.E. (2002). Predictors of Aspiration Pneumonia in Nursing Home Residents. Dysphagia.

[23] Patel D.A., Krishnaswami S., Steger E., Conover E., Vaezi M.F., Ciucci M.R., Francis D.O. (2018). Economic and survival burden of dysphagia among inpatients in the United States. Dis. Esophagus.

[24] Shune S.E., Karnell L.H., Karnell M.P., van Daele D.J., Funk G.F. (2012). Association between severity of dysphagia and survival in patients with head and neck cancer. Head Neck.

[25] Karvonen-Gutierrez C.A., Ronis D.L., Fowler K.E., Terrell J.E., Gruber S.B., Duffy S.A. (2008). Quality of Life Scores Predict Survival Among Patients with Head and Neck Cancer. J. Clin. Oncol..

[26] Jones E., Speyer R., Kertscher B., Denman D., Swan K., Cordier R. (2018). Health-Related Quality of Life and Oropharyngeal Dysphagia: A Systematic Review. Dysphagia.

[27] Eslick G.D., Talley N.J. (2008). Dysphagia: Epidemiology, risk factors and impact on quality of life—A population-based study: DYSPHAGIA—A POPULATION-BASED STUDY. Aliment. Pharmacol. Ther..

[28] Plowman-Prine E.K., Sapienza C.M., Okun M.S., Pollock S.L., Jacobson C., Wu S.S., Rosenbek J.C. (2009). The relationship between quality of life and swallowing in Parkinson’s disease. Mov. Disord..

[29] Cichero J.A. (2013). Thickening agents used for dysphagia management: Effect on bioavailability of water, medication and feelings of satiety. Nutr. J..

[30] Marik P.E. (2001). Aspiration pneumonitis and aspiration pneumonia. N. Engl. J. Med..

[31] Allen J., Greene M., Sabido I., Stretton M., Miles A. (2020). Economic costs of dysphagia among hospitalized patients. Laryngoscope.

[32] Ekberg O., Hamdy S., Woisard V., Wuttge-Hannig A., Ortega P. (2002). Social and Psychological Burden of Dysphagia: Its Impact on Diagnosis and Treatment. Dysphagia.

[33] Namasivayam-MacDonald A., Shune S. (2018). The Burden of Dysphagia on Family Caregivers of the Elderly: A Systematic Review. Geriatrics.

[34] Nund R.L., Scarinci N.A., Cartmill B., Ward E.C., Kuipers P., Porceddu S.V. (2015). Third-party disability in carers of people with dysphagia following non-surgical management for head and neck cancer. Disabil. Rehabil..

[35] Philippot P., Chapelle G., Blairy S. (2002). Respiratory feedback in the generation of emotion. Cogn. Emot..

[36] Boiten F.A., Frijda N.H., Wientjes C.J.E. (1994). Emotions and respiratory patterns: Review and critical analysis. Int. J. Psychophysiol..

[37] Arch J.J., Craske M.G. (2006). Mechanisms of mindfulness: Emotion regulation following a focused breathing induction. Behav. Res. Ther..

[38] Kaushik R.M., Kaushik R., Mahajan S.K., Rajesh V. (2006). Effects of mental relaxation and slow breathing in essential hypertension. Complement. Ther. Med..

[39] Carnaby G.D., Harenberg L. (2013). What is ‘Usual Care’ in Dysphagia Rehabilitation: A Survey of USA Dysphagia Practice Patterns. Dysphagia.

[40] Crary M., Sura L., Madhavan A., Carnaby-Mann G. (2012). Dysphagia in the elderly: Management and nutritional considerations. Clin. Interv. Aging.

[41] Hillemacher T., Gräßel E., Tigges S., Bleich S., Neundörfer B., Kornhuber J., Hecht M. (2004). Depression and bulbar involvement in amyotrophic lateral sclerosis. Amyotroph. Lateral Scler. Other Mot. Neuron Disord..

[42] Chow E.S.L., Kong B.M.H., Wong M.T.P., Draper B., Lin K.L., Ho S.K.S., Wong C.P. (2004). The prevalence of depressive symptoms among elderly Chinese private nursing home residents in Hong Kong. Int. J. Geriatr. Psychiatry.

[43] Santos M., Richards C.S., Bleckley M.K. (2007). Comorbidity between depression and disordered eating in adolescents. Eat. Behav..

[44] Seppälä E.M., Bradley C., Moeller J., Harouni L., Nandamudi D., Brackett M.A. (2020). Promoting Mental Health and Psychological Thriving in University Students: A Randomized Controlled Trial of Three Well-Being Interventions. Front. Psychiatry.

[45] Goldstein M.R., Lewin R.K., Allen J.J.B. (2020). Improvements in well-being and cardiac metrics of stress following a yogic breathing workshop: Randomized controlled trial with active comparison. J. Am. Coll. Health.

[46] Dudley D.L., Glaser E.M., Jorgenson B.N., Logan D.L. (1980). Psychosocial Concomitants to Rehabilitation in Chronic Obstructive Pulmonary Disease. CHEST.

[47] Clayton N.A., Carnaby G.D., Peters M.J., Ing A.J. (2014). Impaired laryngopharyngeal sensitivity in patients with COPD: The association with swallow function. Int. J. Speech Lang. Pathol..

[48] Martin-Harris B., Brodsky M.B., Price C.C., Michel Y., Walters B. (2003). Temporal coordination of pharyngeal and laryngeal dynamics with breathing during swallowing: Single liquid swallows. J. Appl. Physiol..

[49] Martin-Harris B., Brodsky M.B., Michel Y., Ford C.L., Walters B., Heffner J. (2005). Breathing and swallowing dynamics across the adult lifespan. Arch. Otolaryngol. Neck Surg..

[50] Martin-Harris B., McFarland D., Hill E.G., Strange C.B., Focht K.L., Wan Z., Blair J., McGrattan K. (2014). Respiratory-Swallow Training in Patients with Head and Neck Cancer. Arch. Phys. Med. Rehabil..

[51] Martin B.J.W., Logemann J.A., Shaker R., Dodds W.J. (1994). Coordination between respiration and swallowing: Respiratory phase relationships and temporal integration. J. Appl. Physiol..

[52] Cvejic L., Churchward T., Harding R., Turton A., Finlay P., Massey D., Bardin P.G., Guy P. (2011). Laryngeal penetration and aspiration in individuals with stable COPD: Aspiration in COPD. Respirology.

[53] Huff A., Reed M.D., Smith B.K., Brown E.H., Ovechkin A.V., Pitts T. (2018). Strategies for the Integration of Cough and Swallow to Maintain Airway Protection in Humans. Lung.

[54] Hopkins-Rossabi T., Curtis P., Temenak M., Miller C., Martin-Harris B. (2019). Respiratory Phase and Lung Volume Patterns During Swallowing in Healthy Adults: A Systematic Review and Meta-Analysis. J. Speech Lang. Hear. Res..

[55] Drulia T. The Effects of Lung Volume on Swallowing in Chronic Obstructive Pulmonary Disease. https://commons.lib.jmu.edu/cgi/viewcontent.cgi?article=1134&context=diss201019.

[56] Good-Fratturelli M.D., Curlee R.F., Holle J.L. (2000). Prevalence and nature of dysphagia in va patients with copd referred for videofluoroscopic swallow examination. J. Commun. Disord..

[57] Mokhlesi B., Logemann J.A., Rademaker A.W., Stangl C.A., Corbridge T.C. (2002). Oropharyngeal deglutition in stable COPD. CHEST.

[58] Coelho C.A. (1987). Preliminary findings on the nature of dysphagia in patients with chronic obstructive pulmonary disease. Dysphagia.

[59] Stein M., Williams A.J., Grossman F., Weinberg A.S., Zuckerbraun L. (1990). Cricopharyngeal dysfunction in chronic obstructive pulmonary disease. CHEST.

[60] Kobayashi S., Kubo H., Yanai M. (2007). Impairment of the swallowing reflex in exacerbations of COPD. Thorax.

[61] Terada K., Muro S., Ohara T., Kudo M., Ogawa E., Hoshino Y., Hirai T., Niimi A., Chin K., Mishima M. (2010). Abnormal Swallowing Reflex and COPD Exacerbations. CHEST.

[62] Steidl E., Ribeiro C., Gonçalves B., Fernandes N., Antunes V., Mancopes R. (2014). Relationship between Dysphagia and Exacerbations in Chronic Obstructive Pulmonary Disease: A Literature Review. Int. Arch. Otorhinolaryngol..

[63] Schroedl C.J., Yount S.E., Szmuilowicz E., Hutchison P.J., Rosenberg S.R., Kalhan R. (2014). A Qualitative Study of Unmet Healthcare Needs in Chronic Obstructive Pulmonary Disease. A Potential Role for Specialist Palliative Care?. Ann. Am. Thorac. Soc..

[64] Seamark D.A., Blake S.D., Seamark C.J., Halpin D.M. (2004). Living with severe chronic obstructive pulmonary disease (COPD): Perceptions of patients and their carers: An interpretative phenomenological analysis. Palliat. Med..

[65] Schane R.E., Woodruff P.G., Dinno A., Covinsky K.E., Walter L.C. (2008). Prevalence and Risk Factors for Depressive Symptoms in Persons with Chronic Obstructive Pulmonary Disease. J. Gen. Intern. Med..

[66] Vögele C., Von Leupoldt A. (2008). Mental disorders in chronic obstructive pulmonary disease (COPD). Respir. Med..

[67] Yohannes A.M., Müllerová H., Hanania N.A., Lavoie K., Tal-Singer R., Vestbo J., Rennard S.I., Wouters E.F., Mülerova H. (2016). Long-term course of depression trajectories in patients with COPD: A 3-year follow-up analysis of the evaluation of COPD longitudinally to identify predictive surrogate endpoints cohort. CHEST.

[68] Atlantis E., Fahey P., Cochrane B., Smith S. (2013). Bidirectional Associations Between Clinically Relevant Depression or Anxiety and COPD. CHEST.

[69] von Leupoldt A., Taube K., Henkhus M., Dahme B., Magnussen H. (2010). The impact of affective states on the perception of dyspnea in patients with chronic obstructive pulmonary disease. Biol. Psychol..

[70] Iyer A.S., Bhatt S.P., Garner J.J., Wells J.M., Trevor J.L., Patel N.M., Kirkpatrick D., Williams J.C., Dransfield M.T. (2015). Depression is Associated with Readmission due to Acute Exacerbation of Chronic Obstructive Pulmonary Disease. Ann. Am. Thorac. Soc..

[71] Yohannes A.M., Kaplan A., Hanania N.A. (2018). Anxiety and Depression in Chronic Obstructive Pulmonary Disease: Recognition and Management. Cleve. Clin. J. Med..

[72] Nguyen N.P., Frank C., Moltz C.C., Vos P., Smith H.J., Karlsson U., Dutta S., Midyett A., Barloon J., Sallah S. (2005). Impact of dysphagia on quality of life after treatment of head-and-neck cancer. Int. J. Radiat. Oncol..

[73] McHorney C.A., Robbins J., Lomax K., Rosenbek J.C., Chignell K., Kramer A.E., Bricker D.E. (2002). The SWAL-QOL and SWAL-CARE Outcomes Tool for Oropharyngeal Dysphagia in Adults: III. Documentation of Reliability and Validity. Dysphagia.

[74] Mintz S.W., Bois C.M.D. (2002). The Anthropology of Food and Eating, Annu. Rev. Anthropol..

[75] Plastow N.A., Atwal A., Gilhooly M. (2014). Food activities and identity maintenance in old age: A systematic review and meta-synthesis. Aging Ment. Health.

[76] Perry L., McLaren S. (2003). Coping and adaptation at six months after stroke: Experiences with eating disabilities. Int. J. Nurs. Stud..

[77] Klinke M.E., Wilson M.E., Hafsteinsdóttir T.B., Jónsdóttir H. (2013). Recognizing new perspectives in eating difficulties following stroke: A concept analysis. Disabil. Rehabil..

[78] Ford E.S., Murphy L.B., Khavjou O., Giles W.H., Holt J.B., Croft J.B. (2015). Total and State-Specific Medical and Absenteeism Costs of COPD Among Adults Aged 18 Years in the United States for 2010 and Projections Through 2020. CHEST.

[79] Rogus-Pulia N.M., Rusche N., Hind J.A., Zielinski J., Gangnon R.E., Safdar N., Robbins J. (2016). Effects of Device-Facilitated Isometric Progressive Resistance Oropharyngeal Therapy on Swallowing and Health-Related Outcomes in Older Adults with Dysphagia. J. Am. Geriatr. Soc..

[80] Verdonschot R.J., Baijens L.W., Vanbelle S., van de Kolk I., Kremer B., Leue C. (2017). Affective symptoms in patients with oropharyngeal dysphagia: A systematic review. J. Psychosom. Res..

[81] Verdonschot R.J., Baijens L.W., Serroyen J.L., Leue C., Kremer B. (2013). Symptoms of anxiety and depression assessed with the Hospital Anxiety and Depression Scale in patients with oropharyngeal dysphagia. J. Psychosom. Res..

[82] Patterson J.M., McColl E., Carding P.N., Hildreth A.J., Kelly C., Wilson J.A. (2014). Swallowing in the first year after chemoradiotherapy for head and neck cancer: Clinician-and patient-reported outcomes: Swallowing Outcomes after Chemoradiotherapy for Head and Neck Cancer. Head Neck.

[83] Leder S.B., Suiter D.M., Agogo G.O., Cooney L.M. (2016). An Epidemiologic Study on Ageing and Dysphagia in the Acute Care Geriatric-Hospitalized Population: A Replication and Continuation Study. Dysphagia.

